# Prior Experience but Not Size of Error Improves Motor Learning on the Split-Belt Treadmill in Young Children

**DOI:** 10.1371/journal.pone.0093349

**Published:** 2014-03-27

**Authors:** Susan K. Patrick, Kristin E. Musselman, Junichi Tajino, Hsiu-Chung Ou, Amy J. Bastian, Jaynie F. Yang

**Affiliations:** 1 Department of Physical Therapy and Centre for Neuroscience, University of Alberta, Edmonton, Alberta, Canada; 2 Kennedy Krieger Institute and Johns Hopkins School of Medicine, Baltimore, Maryland, United States of America; University of California, Merced, United States of America

## Abstract

Children can modify learned motor skills, such as walking, to adapt to new environments. Movement errors in these new situations drive the learning. We used split-belt walking to determine whether size of the error affects the degree of learning. Twenty-two children (aged 2–5 y) walked on the split-belt treadmill on two separate days spaced 1 week apart. Twenty-eight adults served as controls. On Day 1, children experienced an abrupt change in belt speeds (from 1∶1 to 2∶1 differential) resulting in large errors, or a gradual change (same change in speed over 12–15 min), resulting in small errors. Learning was measured by the size of the aftereffect upon return to a 1∶1 differential. On Day 2 (1 week later), the leg on the fast belt was reversed, as was the method of introducing the speed differential. We found that the error size did not affect learning. Unexpectedly, learning was greater on Day 2 compared to Day 1, especially for children under 4 y of age, despite the fact that the task was opposite to that of Day 1, and did not influence learning in adults. Hence, 11 additional children under 4 y of age were tested with belts running at the same speed on Day 1, and with a 2∶1 speed differential (abrupt introduction) on Day 2. Surprisingly, learning was again greater on Day 2. We conclude that size of error during split-belt walking does not affect learning, but experience on a treadmill does, especially for younger children.

## Introduction

Young children are constantly modifying previously learned motor skills, such as walking, in response to changes in the environment as well as to growth and development. The modification of a well-established movement in response to a predictable change is called motor adaptation [1]. This learning process is error-driven and cerebellum-dependent [1], [2]. Despite the frequency at which children modify motor skills, little is known about motor adaptation in children.

Recently, the split-belt treadmill has been used to study motor adaptation in children as young as 8 months old [3]. A split-belt treadmill has two belts (one for each leg) that can move at different speeds. When one walks with one belt moving faster than the other, spatial and temporal asymmetries in gait result [4]. After several minutes of split-belt walking, healthy adults adapt their walking pattern such that symmetry is restored. When adults return to walking under normal conditions (i.e. belts moving at the same speed), called tied-belt, they again show asymmetries in their gait that are opposite in direction to those shown when first walking on the split-belt treadmill. These aftereffects indicate that a new motor pattern has been acquired and stored [5]. Likewise, children also adapt their gait when walking on a split-belt treadmill and show aftereffects [3], [6], despite ongoing cerebellar development throughout the first decade of life [7]–[9]. However, adaptation in the spatial domain is seldom seen in children younger than 3 years of age [3] and even in older children, the time course of spatial adaptation is slow compared with adults [6].

Previously we observed that young children showed greater learning (i.e., aftereffects) in the spatial domain during split-belt walking if the size of the error (i.e., spatial asymmetry) driving the adaptation was small [3]. Smaller errors also resulted in larger aftereffects [10], [11] and greater retention [12], [13] in uninjured adults performing reaching movements under different types of persistent perturbation (visuomotor rotation [10]; gain change in cursor [11]; force field [12], [13]). Similarly, adults with cerebellar damage learned better with small errors when performing reaching movements in a force-field [14], but these results were not replicated and were later attributed to a confound related to force-field direction [15]. Contrasting results have also been reported, i.e., no effect of error size in adults with visuomotor rotation [16], and better learning with large errors in children with Developmental Coordination Disorder (DCD) performing drawing movements with visuomotor rotation [17].

Small errors also facilitate learning of motor and non-motor *skills* in healthy children and those with disabilities [18], [19]. Motor skill learning is defined here as learning that results in an improvement in the motor skill either in accuracy or speed above baseline performance [20], distinct from motor adaptation that returns performance to baseline. In the case of overhand throwing (motor skill learning), children with mild intellectual disability benefitted the most from training that involved gradually increasing the difficulty of the task, and hence keeping the motor errors small, over the course of a training session [21]. Minimizing error during learning is believed to engage implicit learning strategies and reduce the amount of cognitive processing involved in the learning process [22].

Do small errors during practice, which leads to greater learning of motor skills, also result in greater learning of a motor adaptation task? Here we sought to systematically test whether young children show greater learning (i.e., aftereffects) after a single session of spilt-belt walking if the size of the error driving the learning is small. Small errors (i.e., gait asymmetries) were induced by gradually increasing the speed differential of the treadmill belts from 1∶1 to 2∶1 (called the gradual condition). Larger errors occurred when a 2∶1 speed differential was introduced immediately (called the abrupt condition). We hypothesized that the children would show greater learning following the gradual condition.

## Methods

### Subjects

The study was approved by the Health Research Ethics Board, University of Alberta and Alberta Health Services, Pro00003878. All experiments were performed with the written, informed consent of adult subjects and of parents of child subjects, in accordance with the Declaration of Helsinki Guidelines on Human Experimentation and with the approval of the above ethics board. Data for this investigation were obtained from a total of 33 children aged 2–5 y, and 28 adults. Children were recruited from New Mothers' groups in local public health clinics, advertisements in a free locally published magazine, through a university listserve, and by word of mouth. Adults were recruited using posters on campus, and by word of mouth.

### Experimental protocol

**Figure 1:**
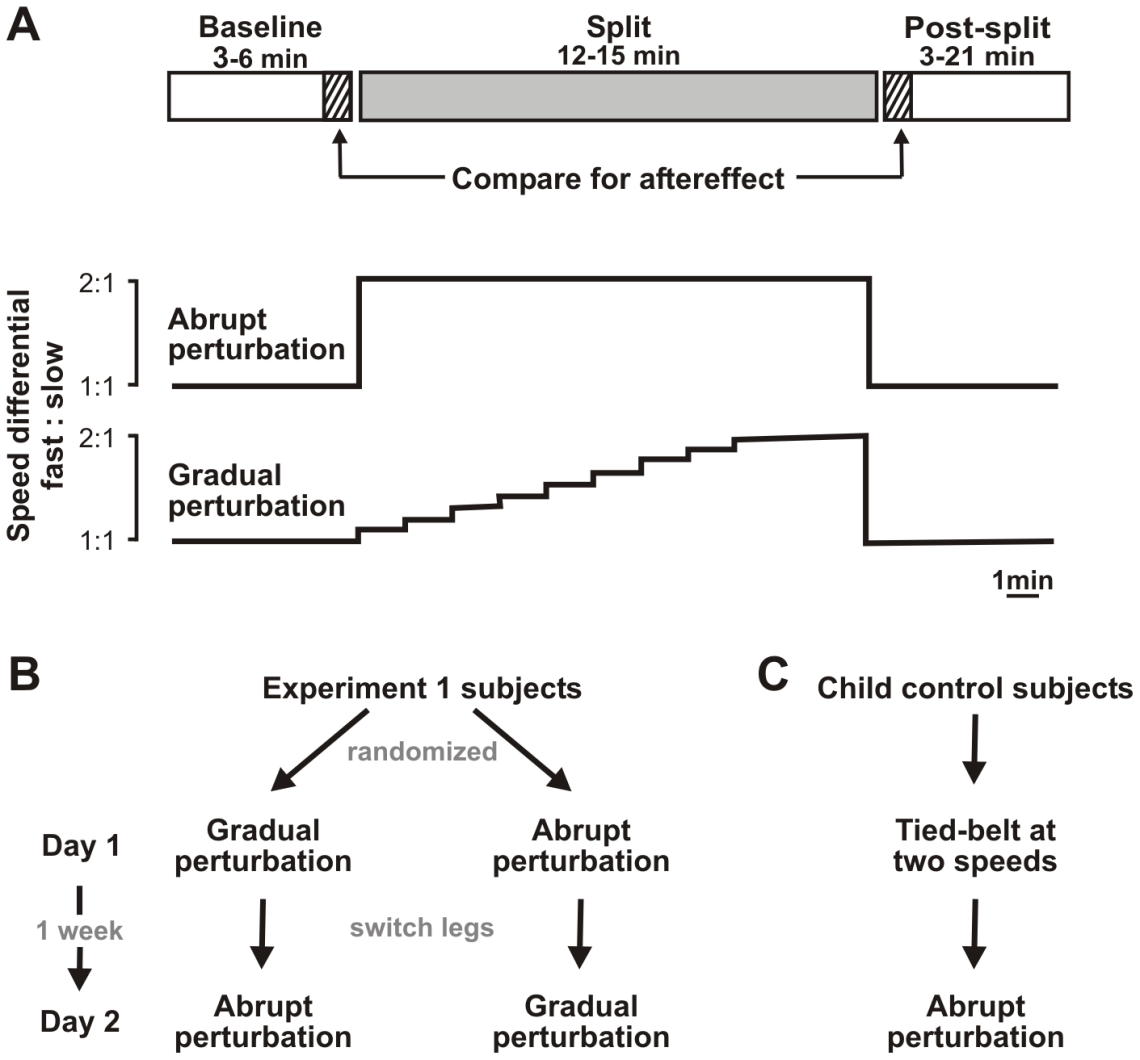
Experimental protocol and design. **A.** Top graph: Schematic of split-belt testing paradigm. During a single testing session, subjects experienced tied-belt walking for 3–6 minutes (Baseline), followed by 12–15 min period of split-belt walking (Split), and a final period of tied-belt walking (Post-split). Durations of periods were determined a priori (see text). Post-split duration ranged from 3–6 minutes for children, but was extended to 21 minutes for adult subjects. The hatched bars indicate the time points of the steps used for calculation of aftereffect. Bottom graph: Two methods of introducing the 2∶1 belt speed differential: abruptly changing the fast belt, or gradually increasing the fast belt speed in increments of 0.045 m/s over the first ¾ of the split-belt period, then maintaining the 2∶1 ratio for the final ¼ of the split-belt period. **B.** Subject allocation to the two sequences (abrupt or gradual) of testing in Experiment 1. The leg on the fast belt was switched between Day 1 and Day 2. C. Child control subjects (Experiment 2) were exposed to tied-belt walking at two speeds on Day 1, and to split-belt walking (abrupt perturbation) on Day 2.

The split-belt paradigm was used as a model of motor adaptation, and consisted of three periods of walking: baseline, split, and post-split (Fig. 1A). The duration of the baseline for children was dependent on the quality of the walking (i.e., sufficient number of steps without jumping, playing … etc.). The duration of the adaptation and post periods for the children were determined a priori, based on our judgement of the likelihood of the child completing the test session. For example, from our interaction with the child prior to the experiment and during baseline walking, we assigned a child to either 12 min or 15 min of split-belt adaptation (i.e., duration of split). Adults had fixed durations for all periods. During the baseline period (3–6 min), subjects walked in the tied-belt condition (treadmill belts running at the same speed). For children, the speed used was based on the length of their leg. For example, if the leg length (distance between the lateral malleolus and the greater trochanter) was 0.4 m, then they walked at 0.4 m/s. Baseline speed was set to 0.5 m/s for all adults. During the split period (12–15 min), the two treadmill belts ran at different speeds, creating a perturbation to walking. Two different perturbations, abrupt and gradual, were applied. An immediate introduction of a 2∶1 speed differential was used to impose an abrupt perturbation: in this case, one of the belts ran at the speed used during baseline (slow speed), and the other ran at twice this speed (fast speed). By contrast, gradually incrementing the speed of one belt throughout most of the split period served as a gradual perturbation: in this case, one belt ran at the slow speed, while the speed of the other increased in increments of 0.045 m/s over the first ¾ of the split period (i.e., ∼0.2 increment in speed differential per min); the belts ran at the full 2∶1 speed differential for the final ¼ of the split period. During the post-split period, the treadmill was returned to the tied-belt condition at the slow speed. The post-split period lasted 3–6 minutes for children. For adults, a longer (21 min) post-split period was used to ensure maximal washout of adaptation. Walking was limited to 3 min per trial, with brief (∼1 min or less) rests between trials.

Children were distracted as much as possible from the walking task using videos, stickers, food, puzzles, and conversation. Adult subjects were distracted through videos.

#### Experiment 1

Children aged 2–5 y and adults were recruited. Since children vary considerably in their maturational rate, a within-subject comparison (i.e., same subject tested under two conditions) would help reduce resulting variability. Further, given that motor learning on the split-belt treadmill shows no transfer or interference between the legs in adults (i.e., left leg fast, right leg slow learning does not interfere with left leg slow, right leg fast learning) [23], we tested each child twice (Day 1 and Day 2), at least one week apart, switching the leg on the fast belt between days (Fig. 1B). On Day 1, children experienced one type of perturbation (abrupt or gradual), and on Day 2, they experienced the other. The order in which the perturbations were applied alternated between children in the order in which they were recruited. We anticipated no differences in the adaptation between Day 1 and Day 2, given the long interval between the days and the switching of the leg on the fast belt.

Adult subjects were tested using the same protocol, with one addition: on Day 1, they were presented with a brief trial (5–10 steps) of the full split condition (2∶1 speed differential) between the two baseline trials. Similar brief exposures to abrupt changes in speed differential have been shown to be effective in eliminating an augmentation in response due to the effects of surprise that may otherwise occur with subsequent abrupt changes [24]. This extra trial was only administered to adults since children were not observed to exhibit a similar effect of surprise.

#### Experiment 2

In Experiment 1, Day 2 aftereffects were unexpectedly greater than those of Day 1 for younger children, but not for older children or adults (see Results). We wondered if this was because older children had more walking experience, and perhaps even prior experience walking on a treadmill, as opposed to experience with the split-belt phenomenon itself. To test the effect of prior experience walking unperturbed on a treadmill, 11 additional children 2–3.9 y of age were recruited to act as controls (henceforth called *child controls*). On Day 1, these children walked in the tied-belt conditions at all times (Fig. 1C). As with the spilt-belt paradigm, baseline and post periods consisted of walking with belts tied at the slow speed. In place of the split period, the children encountered four 3-minute trials of walking at two different speeds: for the first 1.5 minutes of each trial, the treadmill belts ran at a speed 1.5× the slow speed; halfway through each trial, the belts slowed over 1–2 seconds to the slow speed. On Day 2 (1 week later), the children were tested with the split-belt paradigm using an abrupt perturbation.

### Data collection

Most children and all adult subjects walked independently on a Woodway split-belt treadmill (Split-Belt, Woodway USA, Waukesha, WI). One child who was quite tiny and another who was afraid of the adult treadmill were tested instead on a treadmill custom-built for infants and toddlers (model INFSBT-FP, R. Gramlich and S. Graziano, University of Alberta); as children supported their own weight on either treadmill, the specific treadmill used was not expected to affect the results. Children were spotted by a parent or experimenter. Most children and all adults held onto the front bar of the treadmill.

A 3-dimensional motion capture system (Optotrak: 3D Investigator (Edmonton) or Certus (Baltimore), Northern Digital Inc., Waterloo, Ontario) was used to record movement of the legs. Two sets of three infrared cameras tracked (100 s^−1^) infrared emitting markers placed bilaterally on the trunk (lateral midline), hip (greater trochanter), knee (lateral joint line), ankle (lateral malleolus) and foot (head of 5^th^ metatarsal).

For all but three children, trials were also videotaped at 30 frames/s (JVC Everio GZ-MG330, Victor Company of Japan, Ltd.) so that steps where the child tripped, stopped walking, jumped, or was playing on the treadmill could be identified and excluded from analysis. For the three children that were not videotaped, aberrant steps were noted during the experiment and excluded off-line. Adult trials were also videotaped, and the occasional aberrant step removed from analysis.

A push-button produced a 5V analog signal to mark changes in treadmill speed during a gradual perturbation, and during multi-speed trials of Day 1 for child controls. The Optotrak system emitted an analog signal identifying the beginning and end of data collection by the system. This signal and that of a digital counter were used to synchronize Optotrak with video data, and with push-button signals when collected. The digital counter advanced a light-emitting diode display in view of the camera and emitted a 5V pulse every second. All analog signals were digitized at 500 s^−1^ (AxoScope; Molecular Devices, Sunnyvale, CA).

### Data analysis

Data were analyzed off-line using custom-written software (Matlab, The MathWorks). The leg that walked on the faster belt during the split period is referred to as the “fast leg” (even during tied-belt periods). The “slow leg” refers to the leg that walked on the slower belt, and was used as the reference leg for symmetry measures. Although child controls (i.e., Experiment 2) did not have a split period on Day 1, the reference leg (for symmetry measures) was switched from Day 1 to Day 2, for consistency with analysis of data from Experiment 1.

**Figure 2:**
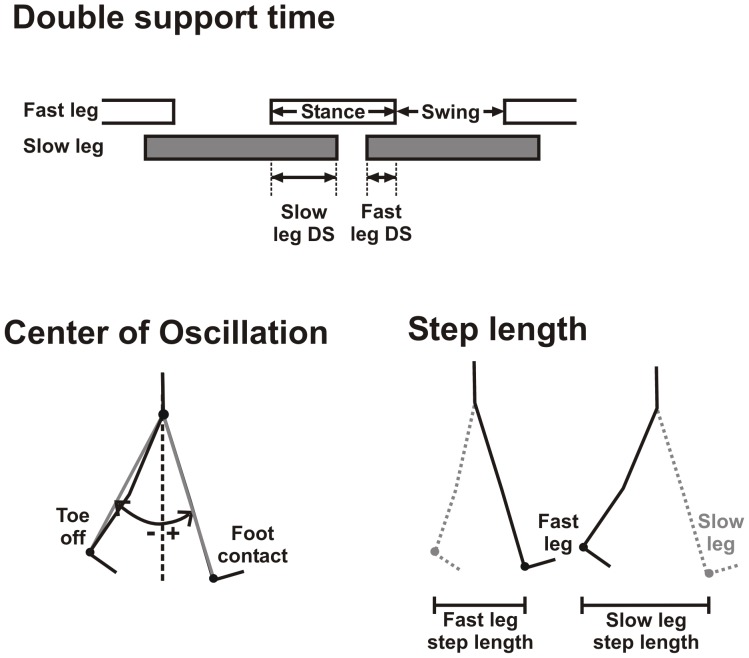
Parameters to determine symmetry of walking. Each walking cycle has two periods of double support (DS), as shown by the overlap between the stance phase of the fast leg (open bars) and the slow leg (filled bars). Slow DS or fast DS is when the slow or the fast leg is trailing, respectively. Symmetry in DS is the difference between the two DS periods, divided by the sum of the two. Centre of oscillation is measured from the limb angles, which is the angle formed with the vertical (dashed line) by the line joining the greater trochanter and lateral malleolus (grey lines). Centre of oscillation for each limb is the midpoint of the limb angle from the start of stance (max limb angle, ∼foot contact) to the end of stance (min limb angle, ∼ toe off). Symmetry in center of oscillation is the difference between centers of oscillation for the fast and slow legs. Step length is the distance between the ankle marker of the leading and trailing limb at the time of foot contact of the leading limb. Symmetry in step length is the difference between the fast and slow step length divided by the sum of the two.

A step was defined as one instance of foot contact to the next instance by the same leg. Limb angle, the angle formed with the vertical by a line connecting greater trochanter and lateral malleolus (Fig. 2), was used to delineate steps: maximum and minimum limb angle approximated foot contact and toe-off, respectively.

Stepping symmetry was quantified using three parameters, calculated for each step for both legs (Fig. 2): double support (a temporal measure), centre of oscillation (a spatial measure), and step length (a measure affected by both temporal and spatial interlimb relationships). These parameters are known to change throughout the split-belt adaptation period in both adults [25], [26] and children [3], [6], [27]. Double support quantifies the duration of time in which both legs are in stance simultaneously. For each step cycle, there are two periods of double support, named for the trailing leg (i.e. fast or slow). Centre of oscillation quantifies the excursion of limb angle travelled by each leg during its stance phase. Step length is the distance between the ankle markers of the two legs at each instance of foot contact, and is named for the leading leg. Symmetry of each parameter was calculated by subtracting the value for the slow leg from that of the fast leg. Double support symmetry was normalized by stride duration, and step length symmetry by the sum of fast and slow step lengths.

To describe changes in step parameters, symmetry during the following four key time points in walking were considered: late baseline, initial split, final split, early post. At each of the four time points, 40 steps were used to describe walking in children, and 10 steps to describe walking in adults. A greater number of steps was chosen for children to accommodate for increased variability and slower time course of change compared to adults [3], [6]. Learning was quantified by calculating an *aftereffect*, the difference in symmetry between late baseline and early post time periods (Fig. 1). *Initial* and *final error* describe changes in stepping observed during the perturbation, and were quantified as the difference in symmetry between late baseline and initial and final split, respectively. Time courses of change were described by averaging every 10 steps for children, and every 3 steps for adults.

### Statistics

Descriptive statistics comprised mean and one standard deviation (SD). Comparative statistics employed are described in the next paragraphs. Parametric tests were used unless data did not pass tests of normality or equal variance. A significant difference was defined as p<0.05, unless otherwise stated.

Statistical comparisons of symmetry in double support, centre of oscillation and step length at key time points *within a day* were achieved using repeated-measures (RM) ANOVA (Bonferroni post-hoc), or Friedman RM ANOVA on Ranks (Tukey post-hoc), if distribution failed normality and/or equal variance test. The following comparisons were made post-hoc: baseline vs. initial split (initial error), baseline vs. final split (final error), baseline vs. early post (aftereffect), and initial vs. final split (adaptation). T-tests or Mann-Whitney rank sum tests were used to compare initial error and aftereffect between abrupt and gradual perturbation types for Day 1.

To determine the effect of perturbation type and day, the aftereffect size from all children in Experiment 1 were compared using a linear mixed-model with two between-subject factors: perturbation type (Abrupt or Gradual), and day of testing (Day 1 or Day 2), and one within-subject factor: sequence of testing (Abrupt first or Gradual first). The analysis first tested for an interaction between perturbation type and day, then for main effects if the interaction was not significant. Adult data were tested in the same way.

Comparison of aftereffect size of child controls (Experiment 2) vs. the younger group of children (aged 2–3.9 y) from Experiment 1 employed independent t-tests and Mann-Whitney rank sum tests; comparisons were made between child controls from Experiment 2, Day 2 with young children in Experiment 1, Day 1, and the same children in Day 2. In addition, the same tests were used to make the same comparisons between data from child controls and data from adults.

In an effort to explore what specifically the children learned on Day 1 to improve learning on Day 2, further analyses were performed on child data from both experiments. Standard deviation of symmetry measures was used as a measure of variability, which may reflect proficiency in the task. For Experiment 1, we used paired t-tests to compare variability at late baseline for Day 1 and Day 2, to determine if there was an improvement in walking proficiency across days. We additionally used Pearson product moment correlations to see if change in variability (from late baseline of Day 1 to the same time period in Day 2) correlated with change in aftereffect from Day 1 to Day 2. For Experiment 2, we concentrated on centre of oscillation data, because this parameter showed the most dramatic change across days: Pearson product moment correlations were employed to find relationships between Day 2 aftereffects and the change in variability from Day 1 late baseline to Day 2 late baseline, to determine if learning was related to improved walking from Day 1 to Day 2.

## Results

Twenty-two children aged 2–5 y (12 female; mean ± SD: 3.9±1.1 y) and 28 adults (15 female; mean ± SD: 26.9±5.8 y) participated in Experiment 1. Eleven additional children (controls) under the age of 4 y (5 female, 2.9±0.5 y) participated in Experiment 2.

### Size of error differs with abrupt and gradual perturbations, but size of aftereffect does not

**Figure 3:**
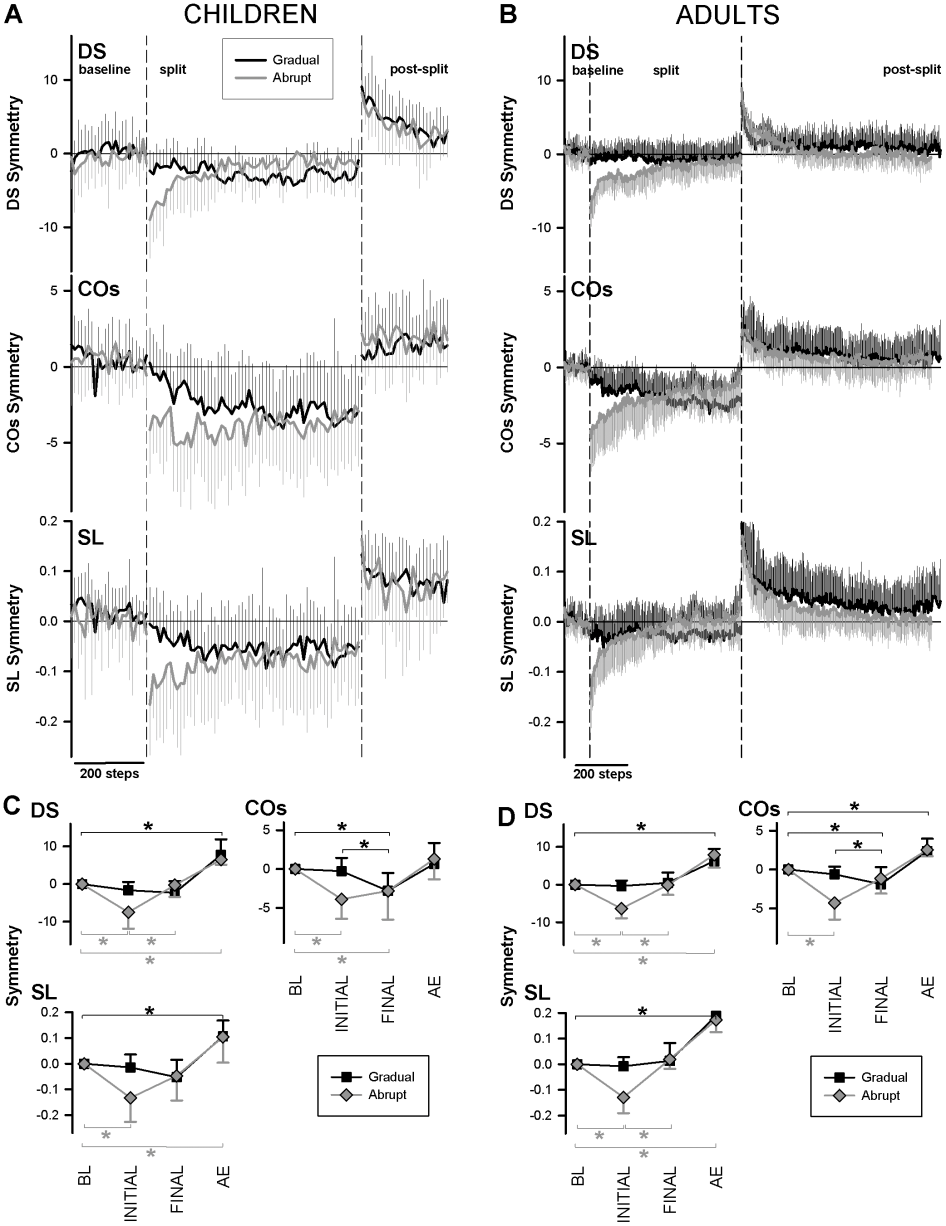
Day 1, Experiment 1: Initial error, but not aftereffect, is affected by method of introduction of perturbation. Top panels: Time courses of double-support time (DS), centre of oscillation (COs) and step length (SL) symmetries (means across subjects, binned), normalized to baseline, for children (**A**) and adults (**B**) for Day 1, Experiment 1. During the split period, the speed differential was introduced gradually (black traces) or abruptly (gray traces). Dashed vertical lines separate the baseline, split-belt and post-split periods of walking. Bins: 10 steps for children, 3 steps for adults; Error bars: 1 SD. Bottom panels: Across subject averages (1 SD error bars) for the four key time periods of baseline (BL), initial split (INITIAL), final split (FINAL), and post-split aftereffect (AE). Each time period is an average of 40 steps for children (**C**) or 10 steps for adults (**D**). *Within* perturbation type, only key statistical comparisons were considered: BL vs. INITIAL, FINAL, and AE; and INITIAL vs. FINAL. Black asterisks indicate statistically significant differences for gradual perturbation, gray for abrupt. Comparisons *between* abrupt and gradual groups were only significant for INITIAL (not shown).

The method of introducing the speed differential between the two treadmill belts (gradual or abrupt) was expected to affect the size of error during the split-belt period, and consequently to affect the size of the aftereffect. Size of error was in fact affected, but size of aftereffect was not. This can be seen in Figure 3, which presents Day 1 mean traces of symmetry measures over baseline, split, and post-split periods for children (A) and adults (B) presented with gradual (black traces) and abrupt (grey traces) introduction of the speed differential. Figure 3C & D are mean symmetry values, across subjects, of initial split (INITIAL), final split (FINAL), and early post periods (AE for aftereffect), normalized to baseline (BL) for clarity.

For both children and adults, abrupt introduction of the speed differential initially produced significant error in all three stepping parameters (Fig. 3C, D, grey * between BL and INITIAL, post-hoc contrasts for DS, SL and COs: t = 6.934, 4.563, 3.777, p<0.001, <0.001, = 0.004 for children, and t = 6.460, 7.831, q = 4.868 (Tukey), p<0.001, <0.001, <0.05 for adults). By contrast, initial error was not significant if the perturbation was introduced gradually. Similarly, initial error was significantly larger for the abrupt compared to gradual perturbation (for DS, SL, CO, t = 3.965, 3.672, 3.869, p<0.001, = 0.002, <0.001 for children, and t = 7.565, 6.351, U = 7.0, p<0.001 in all contrasts for adults). Thus, gradual introduction of the speed differential was successful in gradually introducing error.

Despite differences in introduction of error, the type of perturbation did not alter the aftereffect size for either children or adults on Day 1 (i.e., aftereffect size [mean±SD] for the global measure of step length was gradual = 0.068±0.084, abrupt = 0.113±0.089 for children, and gradual = 0.167±0.069, abrupt = 0.168±0.046 for adults). With the abrupt perturbation, error decreased over the course of the split period, becoming not significantly different from baseline, indication that adaptation had occurred. The one exception was the measure of centre of oscillation; for this parameter, error remained substantial throughout the split-belt period, especially for children, and there was no significant aftereffect in children (for gradual and abrupt perturbations, t = 0.986, 1.431, p = 1.0, 0.979, respectively), suggesting that adaptation was limited. With the gradual perturbation for the children, error remained unchanged over the split period if adaptation occurred with the gradual change in treadmill speed differential (Fig. 3C, black symbols for double support, step length) or gradually increased if adaptation to the perturbation was more limited (Fig. 3C, black symbols for centre of oscillation). Note that the increase in error during gradual perturbation is because of the increasingly discrepant treadmill speeds between the two sides (i.e., experimental manipulation) while minimal adaptation occurred.

**Figure 4:**
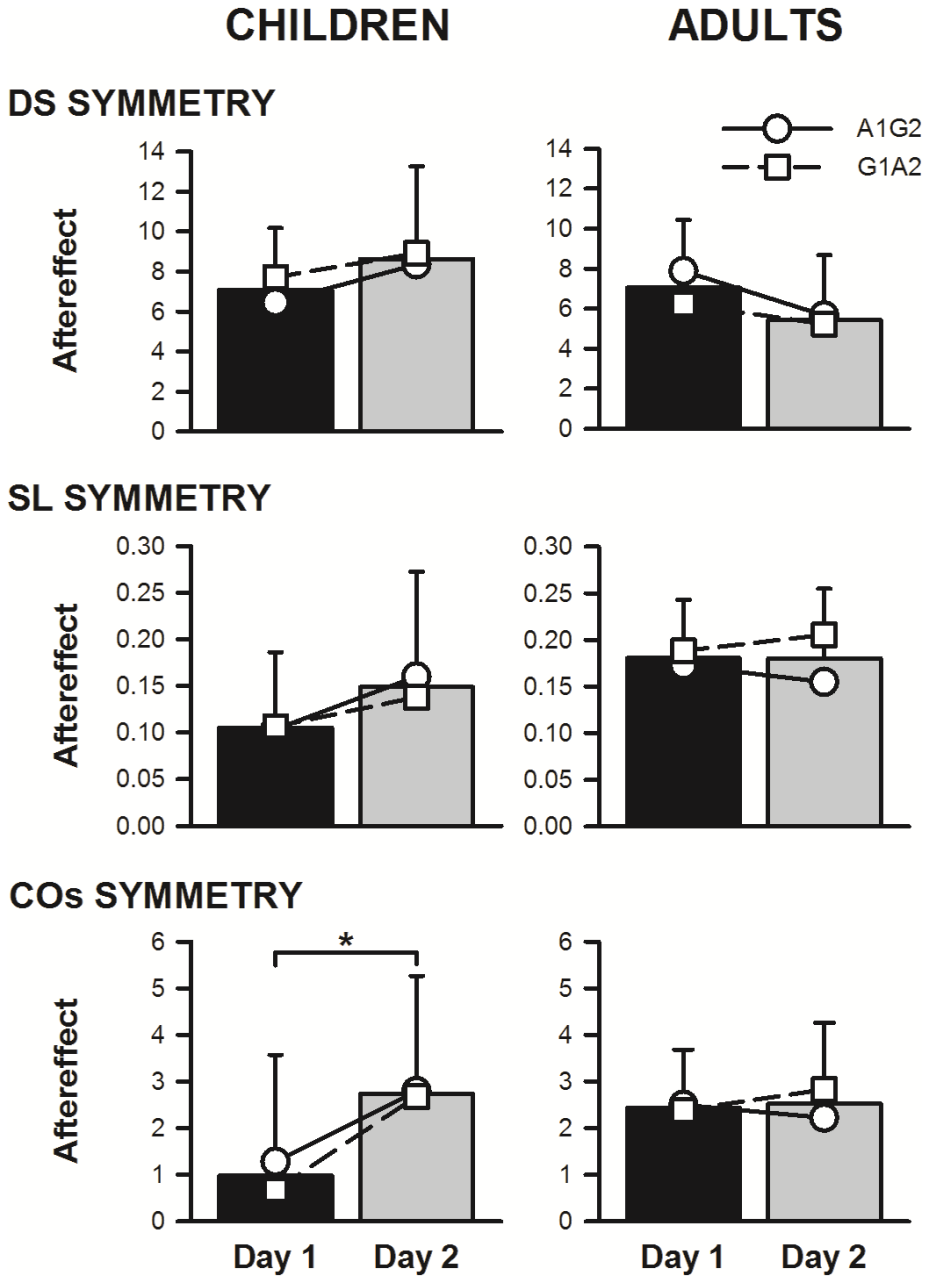
Experiment 1: Children, but not adults, may show greater learning on Day 2. Mean aftereffects for the two exposure sequences: abrupt 1^st^, gradual 2^nd^ (A1G2, circles joined by solid line), and gradual 1^st^, abrupt 2^nd^ (G1A2, squares joined by dashed line) are shown superimposed on the overall mean (bar graph) collapsed across the perturbation type. DS – double support; SL – step length; COs – centre of oscillation. Children showed a significantly larger aftereffect of COs on Day 2 (asterisk), whereas there were no statistical differences between Day 1 and Day 2 for adults. Error bars: 1 SD.

The aftereffect size from Experiment 1 is shown in symbols and lines for each of the participant groups in Figure 4: 1) Day 1 Abrupt, Day 2 Gradual (A1G2 – solid lines and circles), and 2) Day 1 Gradual, Day 2 Abrupt (G1A2 – dashed lines and squares), superimposed on the data collapsed across perturbation type (bar graph). These data were analyzed using a linear mixed-model. The data from children showed a significant main effect of day for centre of oscillation (F = 5.33; p = 0.032; mean±SD: Day 1 = 0.978±0.590, Day 2 = 2.733±2.532), with aftereffect size consistently greater for Day 2 compared to Day 1, but no interaction or main effect for type of perturbation. No differences in any of the contrasts were detected in the adults. Hence, the means by which the speed differential was introduced (gradually or abruptly) resulted in the same level of learning for both children and adults, but children showed increased learning from Day 1 to Day 2, an unexpected finding.

### Younger children, but not older children or adults, showed larger aftereffect on Day 2

**Figure 5:**
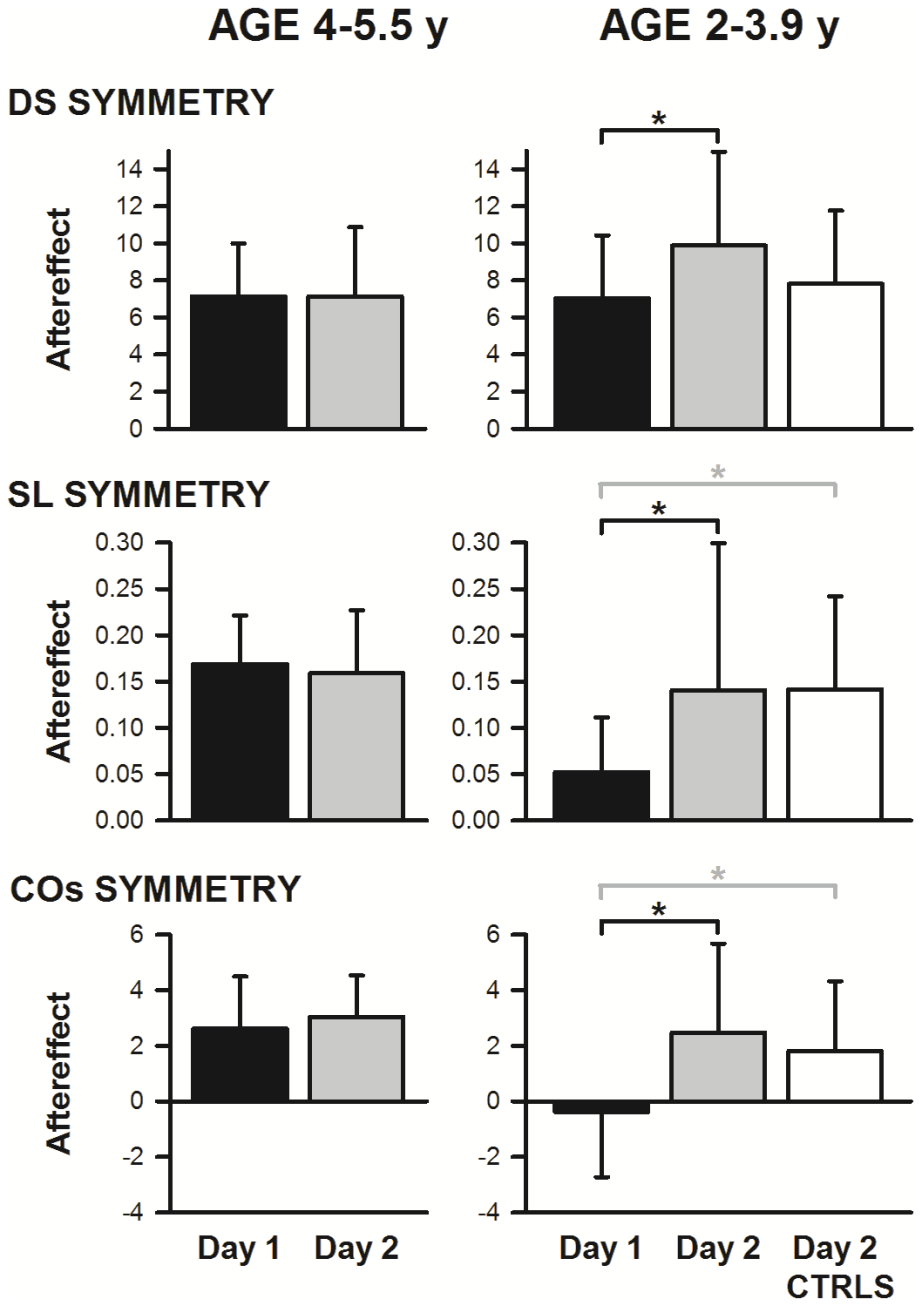
Size of aftereffect was age-dependent. Bars represent mean (+1 SD) aftereffect across children. In Experiment 1, younger children showed differences between Day 1 and Day 2, whereas older children did not (compare black and gray bars; pooled across perturbation type). Child controls of Experiment 2 (CTRLS; all <4.0 y old) walked in the tied-belt condition at two speeds on Day 1, and experienced the abrupt perturbation on Day 2. Day 2 aftereffects for CTRLS (white bars) were comparable to Day 2 aftereffects for the younger children from Experiment 1 who experienced perturbations on both days, and were significantly greater than Day 1 aftereffects for these same children for SL and COs symmetry (asterisks). DS – double support; SL – step length; COs – centre of oscillation.

Since abrupt and gradual introduction of the speed differential resulted in the same aftereffect, we pooled both types of perturbations for comparison of aftereffect across day. Plotting change in aftereffect from Day 1 to Day 2 against age of the child (not shown) revealed a trend (albeit not significant) toward an inverse relationship (r = 0.364). The adult pattern (i.e. no change in aftereffect size from Day 1 to Day 2) seemed to emerge somewhere between the ages of 3.5 and 5.5 years. We thus divided the children into two age groups, using the mid-point of our ages (4 years being the midpoint between 2.5 and 5.5 y): younger (2–3.9 y, 12 children) and older (4–5.5 y, 10 children). The size of the aftereffect on Day 2 was significantly larger than on Day 1 for double support, step length, and centre of oscillation for the younger group, but not for the older group (see Fig. 5; mean±SD, t and p unless otherwise indicated: for young group DS Day 1 = 7.040±3.390, Day 2 = 9.885±5.062, t = −2.274, p = 0.044; SL [median, 25%, 75%]: Day 1 = 0.052, 0.01, 0.1, Day 2 = 0.125, 0.039, 0.168, Z = 2,04 (Wilcoxon sign rank test), p = 0.042; COs Day 1 = −0.384±2.342, Day 2 = 2.475±3.204, t = −2.728, p = 0.020; for the older group DS Day 1 = 7.132±2.861, Day 2 = 7.132±3.748, t = −0.00004, p = 1.0; SL Day 1 = 0.169±0.053, Day 2 = 0.159±0.068, t = 0.428, p = 0.678; COs Day 1 = 2.611±1.875, Day 2 = 3.042±1.487, t = −0.462, p = 0.655).

The question arose, then, as to the cause of this increase in aftereffect on Day 2. Young children (<3 years of age) have previously been found to have greater variability in their treadmill stepping on Day 1 compared with Day 2 [3]. Variability of stepping, as measured by standard deviation during the last 40 baseline steps, did not decrease from Day 1 to Day 2 (DS: t = 1.236, p = 0.242, SL: t = 0.454, p = 0.654, COs: t = 1.534, p = 0.153). Additionally, the number of steps during the split period was not significantly different between Day 1 and Day 2 (t = 0.361, p = 0.722).

### Tied-belt only walking on Day 1 is sufficient to augment aftereffect to perturbation

To determine if simple exposure to walking on the treadmill increased subsequent motor adaptation, 11 children aged 2.1–3.6 years (2.9±0.5 y) acted as child controls. On Day 1, they walked only in the tied-belt condition, at two speeds. On Day 2, they experienced an abrupt change in speed differential between the two belts. Aftereffects on Day 2 in these control children were similar to those observed on Day 2 for the 12 younger children of Experiment 1 (Fig. 5, right panel, compare gray and white bars, U = 49, 56, t = 0.547, p = 0.310, 0.559, 0.590, for DS, SL, COs, respectively; Day 2 Controls [median, 25%, 75%] DS 8.964, 3.864, 10.620, SL 0.103, 0.066, 0.200, [mean±SD] COs 1.815±2.514). Further, in Experiment 1, only 25% of the young children demonstrated significant aftereffects in centre of oscillation on Day 1. On Day 2, the number rose to 50%; this is comparable to the 54% of child controls showing significant aftereffects with their first exposure to the split-belt paradigm. It is also noteworthy that aftereffects on Day 2 in child controls were not significantly different from those observed in the adult subjects for any parameter. Size of aftereffect in the child controls did not correlate with age of subject (Pearson product moment correlation r = −0.365, 0.397, 0.072, p = 0.27, 0.23, 0.81, for DS, SL and COs).

**Figure 6:**
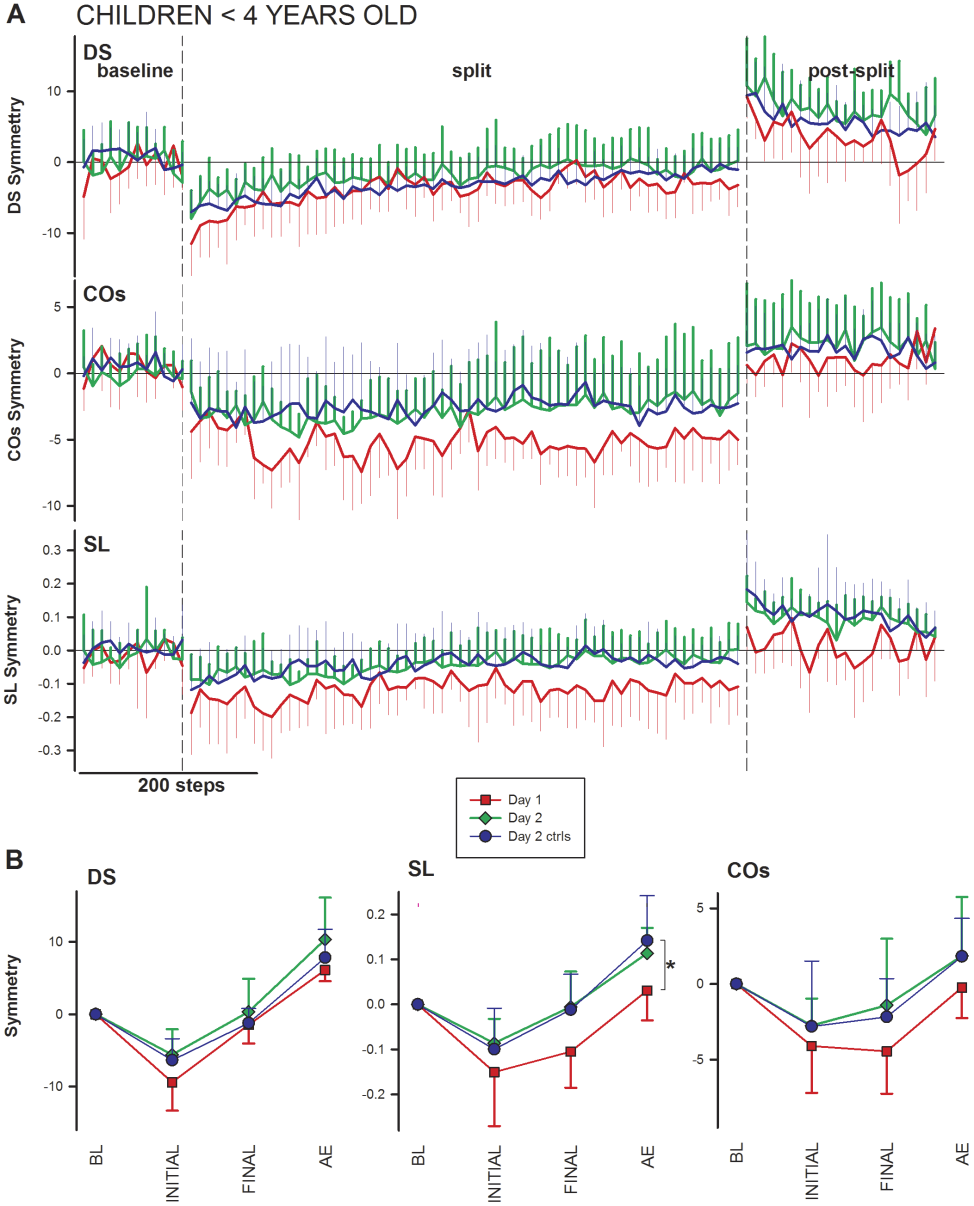
Exposure to tied-belt walking, without perturbation, augments split-belt learning on Day 2 in children <4 y of age. **A.** Time courses of walking symmetry in children <4 y of age, abrupt perturbations only: Ten-step averages are shown for the young children in Experiment 1, Day 1 (i.e., those experiencing an abrupt perturbation 1^st^; red), Day 2 (i.e., those experiencing an abrupt perturbation 2^nd^; green), and the child controls in Experiment 2, Day 2 (blue). The convention of the graph is identical to that of Figure 3. DS – double support; COs – centre of oscillation; SL – step length. **B.** Comparison of Day 2 control data to Experiment 1, Day 1 and Day 2 data: Symbols represent forty-step averages for the same data as shown in A, for the four key time periods: baseline (BL), initial split (SPLIT), final split (FINAL), and post-split aftereffect (AE). Significant differences (asterisk) were found in the SL aftereffect between Day 2 controls and Day 1 of Experiment 1. (Comparisons within groups are not shown.)

Figure 6 presents time courses of double support, centre of oscillation, and step length symmetries for children experiencing an *abrupt* perturbation: on Day 1 with no prior experience (n = 6, red lines; Experiment 1), on Day 2 following gradual perturbation on Day 1 (n = 6, green lines; Experiment 1), or on Day 2 following only tied-belt walking on Day 1 (n = 11, blue lines; Experiment 2). Data from child controls in Experiment 2 fall intermediate to Day 1 and Day 2 of Experiment 1 for double support, but, remarkably, closely resemble Day 2 for centre of oscillation and step length.

We examined several aspects of the centre of oscillation data (because it showed the greatest effects) in attempt to determine what, specifically, the children learned during tied-belt walking on Day 1 that enhanced aftereffects on Day 2. Did they learn to walk more symmetrically as a result of the tied-belt walking? Whereas some children did in fact show a change (improvement or worsening) in symmetry from baseline of Day 1 to baseline of Day 2, there was no apparent relationship to Day 2 aftereffect (correlation: r = 0.155). Similarly, Day 2 aftereffect did not correlate with the level of symmetry in late baseline of Day 2 (correlation: r = 0.083).

## Discussion

Using the split-belt treadmill paradigm, we tested the effects on motor adaptation of perturbation type and of prior exposure to perturbation in 22 children and 28 adults. The type of perturbation (gradual or abrupt introduction of speed differential) had no effect on the aftereffect in either group. The aftereffect, however, tended to be greater on Day 2 compared to Day 1 for children, despite switching which leg walked on the fast belt from one day to the next. This was true of children under 4 y of age only. To determine if exposure to simply walking on a treadmill (with belts tied) affected adaptation on Day 2, 11 additional children under 4 years of age were tested with an abrupt change in speed differential on Day 2, following walking in only the tied belt condition on Day 1. Surprisingly, aftereffects on Day 2 for these control subjects were comparable to those of Day 2 of the original protocol; tied-belt walking on Day 1 was sufficient to augment the size of Day 2 aftereffects.

### Error size during learning

Small movement errors have been suggested to improve learning of motor skills in children [18], [19] and adaptation of reaching movements in adults under visuomotor rotation [10] or in force fields [14]. Our results, however, show that small errors do not improve learning in split-belt walking in children. In this respect, our data is congruent with that of Torres-Oviedo & Bastian [28], who also did not find larger aftereffects with small error compared to larger error during split-belt walking in healthy adults, and others who have failed to find a difference in individuals with cerebellar lesions [15], [16]. Torres-Oviedo & Bastian [28] in fact showed *smaller* aftereffects in healthy adults during catch trials after gradual compared to abrupt introduction of split-belt speed differential [28], whereas in the current study we show no effect of perturbation type. The most likely reason for this discrepancy is the difference in the amount of time the full perturbation was experienced by the gradual group (5 min in current study vs. <1 min in 2012 study). Additional reasons may include the number of subjects, the method of introducing the speed differential: incrementing the fast belt speed only (current study) vs. incrementing the fast and decrementing the slow simultaneously in [28], and the number of steps included in the calculation of the aftereffect size in adults: 10 in the current study and 5 in [28]. Reanalysis of the aftereffect using 5 steps with the adults, however, did not change the results.

Smaller compared to larger movement errors during motor adaptation of arm movements have also been associated with greater retention [12], [13] and greater transfer of learning to similar movements outside of the trained space [29], or to the untrained arm [30]. This is thought to be related to how we attribute the error, i.e., to oneself (small errors), or to the environment/device (large errors) [29]. Errors attributed to oneself are more likely to be generalized to other movements. These differences in generalization suggest that the neural substrates for motor adaptation to small vs large errors may be different [31], and is worth pursuing in the future, particularly with respect to training of walking after injury, when transfer to other related walking environments is highly desirable [32].

### Although limited, children can demonstrate spatial adaptation

Previous work has suggested that very young children may adapt temporal but not spatial interlimb relationships in split-belt walking [3], [6]. Here, however, we establish that some children as young as 2.1 y of age can in fact show adaptation in centre of oscillation and step length. In the current study, 25% of children under 4 y of age experiencing an abrupt or gradual perturbation on Day 1 demonstrated a significant aftereffect in centre of oscillation. This value rose to around 50% of children on Day 2, whether or not a perturbation was experienced on Day 1. These findings are in variance with those of [6], which found no significant aftereffects in centre of oscillation for any of their 10 children aged 3–5 y. A possible reason for this discrepancy is the use of only 3 post-split steps to measure aftereffect in the Vasudevan study compared to 40 steps in the current study. In support of this, we have seen individual cases in which initial error or aftereffect are not manifest until after the first several steps of the time period in question. Thus, taking a larger sample of post-split steps may give a more accurate picture of the ability of young children to adapt. This larger sample is in fact appropriate given the variability of stepping and the slow time course of adaptation and deadaptation of centre of oscillation in children. Despite the discrepancies, both [6] and the current study agree that spatial adaptation does appear to be limited for younger children.

### Neural substrates for motor adaptation in walking

The split-belt paradigm for studying adaptation in walking is unique in that the temporal and spatial aspects of symmetry can be dissociated. The differential maturation of learning temporal vs spatial symmetry in walking suggests different neural mechanisms and/or substrates may be involved. This is further supported by a) the ability of adults to dissociate these two aspects of learning in walking [33], b) the distinct deficits in temporal and spatial learning revealed by cerebellar [2] vs cerebral lesions [27], c) the independent adaptation of the two domains, which can be in opposite direction to each other in people with stroke [34]. The cerebellum has long been considered an important site for the formation and storage of internal models that drive motor adaptation [35]–[37], including walking [38], [39]. Indeed, the cerebellum has access to information regarding both foot contact [40] and limb angle [41]. It remains unclear, however, which parts of the cerebellum, and which other neural substrates with which it interacts, are involved in adaptation of spatial and temporal symmetry in walking. Since the lateral cerebellum is latest to mature [9], we speculate that it may be partly responsible for adapting spatial symmetry of walking in children, which also matures late.

Control of walking symmetry likely involves many other substrates in the nervous system besides the cerebellum, such as central pattern generators (CPGs) of the spinal cord (reviewed in [42]), brainstem centres (reviewed in [43]), and the motor cortex (reviewed in [44]). We presume that cerebellar circuits are critical to enabling the adaptation of symmetry in walking by their interaction with these other centres. For example, reciprocal leg movements in walking might be imagined to involve a minimum of two mutually inhibitory spinal oscillators, one for each leg, as first proposed by Graham Brown [45], [46]. Descending cerebellar input could modify the gains in spinal circuits to achieve either spatial or temporal adaptation, perhaps by altering input gains from sensory signals in the periphery to one or both oscillators. Spatial symmetry, for instance, could be restored by allowing the leg on the fast belt to extend further before swing is initiated, and to flex further before stance is initiated (see limb angle changes in [4] Fig. 7A). Timing symmetry, on the other hand, could be restored by changing the gains between the timing component of the two oscillators. These gain changes might indeed work well with a multilayer CPG, as proposed by McCrea and others, with one layer responsible for timing and another for patterning [47].

The learning we describe here is likely very different from other forms of motor learning during walking. For example, toddlers very quickly learn the coordination of joints within a limb to exhibit the planar covariation of leg segments during walking (i.e., a form of spatial learning) [48], and take much longer to learn the inverted pendulum model of walking [49], which includes the timing of the rise and fall of the centre of gravity and the speed of progression in walking. Thus, the maturation of timing and spatial aspects of motor learning in one task, such as within limb coordination, cannot be extrapolated to other forms of learning in a similar task, such as between limb coordination. The neural substrates responsible for each are likely distinct.

### Children show greater learning with second exposure to treadmill

The most surprising finding is that one day of exposure to the treadmill, with or without a perturbation, improved learning, as exemplified by greater aftereffects on Day 2. These greater aftereffects cannot be explained by transfer of learning, since the leg on the fast belt on Day 2 was opposite that on Day 1; if anything, one would expect interference on Day 2 (indicated by slower adaptation). Further, the two testing days were separated by one week and extensive walking; baseline measures showed no recall (i.e., residual asymmetry in the first few steps), which might be expected if there was retention of aftereffects from Day 1.

Greater learning on Day 2 in young children can neither be explained by differences in the duration of the adaptation period for young children and for older children and adults. While young children experienced a 12-minute adaptation period, children in the older group had exposure times of 12 or 15 min; there were no differences between the older children who experienced 12 min compared to those who experienced 15 min of split-belt walking.

Improved adaptation on Day 2 might be explained by *structure learning*, defined generally as ‘reducing the dimensionality of space that the learning organism has to search to adapt to novel tasks’ [50]. Walking on a treadmill may be an especially novel task for young children, who have had limited experience in walking on different terrain or under different conditions. A single experience of walking on a treadmill may have allowed them to explore the ‘internal parameters’ that map sensory input to motor output specific to treadmill walking. For example, treadmill walking is constrained by the belt speed, so a short step on one leg requires a longer step on the following step of the opposite leg, to prevent ‘drifting’ on the treadmill. Our study suggests that the *structure* learned may be very general in young children, such as learning to not to drift on the treadmill, and does not require the specific experience of a speed differential. In contrast, older children and adults are more likely to have had greater experience in varying walking environments that has allowed them to form useful *structures* for split-belt adaptation, such that an extra day of experience with split-belt walking does not enhance learning on a subsequent day.

### Slow learning rates in children

Despite the increase in aftereffect on Day 2 compared to Day 1 for young children, the rate of learning remained very slow (Fig. 6A). Qualitatively, the rate of adaptation was indistinguishable between the two days, especially for centre of oscillation and step length. These results indicate considerable immaturity in motor adaptation in children; indeed, adaptation of centre of oscillation may not become fully mature until after 12 y of age [6].

Several lines of evidence suggest that the slower rate of adaptation in children is likely also related to immaturity of the cerebellum. First, the time course of motor adaptation in a variety of tasks in adults, including split-belt walking and reaching in force-fields, shows a fast (i.e., rapid reduction in error within the first 10 steps/reaches) and a slow process (i.e., error reduction over tens to hundreds of steps/reaches) [51], [52]. The rapid process is likely cerebellar-dependent, because people with diffuse and severe cerebellar damage show impaired fast learning [1], [2], [14], [53], [54]. The slow process may be more dependent on the cerebrum, as adults with stroke or children with hemispherectomy show deficits in the slow phase of adaptation [27], [52]. Young children show an absence of the rapid process [3], [6], which we speculate may be a result of immaturity of the cerebellum [9]. Indeed, the rate of split-belt adaptation can be altered by transcranial direct currect stimulation over the cerebellum in a polarity-specific way [39].

In conclusion, motor learning in children shows surprising differences from that observed in adults. Not only do different aspects of learning emerge at different ages, but learning (i.e., of split-belt adaptation) can occur very quickly with a single exposure to a related task (i.e., tied-belt treadmill walking). Furthermore, such learning is retained for at least a week without further exposure. The neural mechanisms underlying these interesting phenomena remain to be explored.
